# Improved Influenza Diagnostics through Thermal Contrast Amplification

**DOI:** 10.3390/diagnostics11030462

**Published:** 2021-03-07

**Authors:** Yilin Liu, Li Zhan, Yiru Wang, Joseph Kangas, Daniel Larkin, David R. Boulware, John C. Bischof

**Affiliations:** 1 Department of Mechanical Engineering, University of Minnesota, Twin Cities, Minneapolis, MN 55455, USA; liu00063@umn.edu (Y.L.); zhanx064@umn.edu (L.Z.); wang4370@umn.edu (Y.W.); kanga134@umn.edu (J.K.); 2 HealthEast Grand Avenue Clinic, St. Paul, MN 55105, USA; larkinskemp@me.com; 3 Department of Medicine, Division of Infectious Diseases and International Medicine, University of Minnesota, Twin Cities, Minneapolis, MN 55455, USA; boulw001@umn.edu; 4 Department of Biomedical Engineering, University of Minnesota, Twin Cities, Minneapolis, MN 55455, USA

**Keywords:** influenza, rapid diagnostic test, thermal contrast amplification, point-of-care

## Abstract

Influenza poses a serious health threat and creates an economic burden for people around the world. The accurate diagnosis of influenza is critical to the timely clinical treatment of patients and the control of outbreaks to protect public health. Commercially available rapid influenza diagnostic tests (RIDTs) that are operated by visual readout are widely used in clinics to screen influenza infections, but RIDTs suffer from imperfect analytical sensitivity, especially when the virus concentration in the sample is low. Fortunately, the sensitivity can be simply improved through an add-on signal amplification step, i.e., thermal contrast amplification (TCA). To demonstrate the advantage of TCA for influenza diagnosis, we conducted a prospective cohort study on 345 clinical specimens collected for influenza A and B testing during the 2017–2018 influenza season. All samples were tested using the Quidel QuickVue Influenza A + B test, followed by a TCA readout, and then confirmatory polymerase chain reaction testing. Through the TCA detecting sub-visual weak positives, TCA reading improved the overall influenza sensitivity by 53% for influenza A and 33% for influenza B over the visual RIDTs readings. Even though the specificity was compromised slightly by the TCA protocol (relative decrease of 0.09% for influenza A and 0.01% for influenza B), the overall performance was still better than that achieved by visual readout based on comparison of their plots in receiver operating characteristic space and F1 scores (relative increase of 14.5% for influenza A and 12.5% for influenza B). Performing a TCA readout on wet RIDTs also improved the overall TCA performance (relative increase in F1 score of 48%). Overall, the TCA method is a simple and promising way to improve the diagnostic performance of commercial RIDTs for infectious diseases, especially in the case of specimens with low target analytes.

## 1. Introduction

Influenza, a contagious respiratory illness, poses a continuous severe health threat to people throughout the US and the world. In late 2017, the World Health Organization (WHO) estimated that a total of 250,000 to 500,000 annual deaths were associated with influenza infection based on data over 10 years ago [1]. The annual number of flu-related deaths based on more recent data from a larger, more diverse group of countries increased to 650,000 [1]. In the 2018-2019 influenza season, the Centers for Disease Control and Prevention (CDC) estimated that influenza infection was associated with over 35.5 million illnesses, over 16.5 million medical visits, 490,600 hospitalizations, and 34,200 deaths in the US [2]. In the current pandemic of SARS-CoV-2 (COVID-19), the reported influenza activity in the US and globally is lower than expected, which may be impacted by the COVID-19 pandemic and needs to be interpreted with caution [3,4]. The Global Influenza Surveillance and Response System (GISRS) from the WHO suggests that the threat of influenza epidemics and pandemics persists during the COVID-19 pandemic and that countries are advised to remain vigilant and active in influenza sentinel surveillance when implementing COVID-19 surveillance [5]. Given its high morbidity, influenza has also imposed significant healthcare costs and burdens [6]. A previous study estimated that in 2003, the annual direct medical cost for influenza treatment was approximately $10.4 billion in the US [7], while the average annual cost for seasonal influenza in Italy in the period 1999–2008 was approximately US $1.6 billion [8].

The timely and accurate diagnosis of influenza infection is imperative so that antiviral therapy can be appropriately prescribed, unnecessary testing reduced, nosocomial transmission prevented, and thousands of hospitalizations prevented (especially among children and older adults). While vaccination helps to reduce influenza morbidity and mortality, the vaccine effectiveness varies from 11–69% year-to-year [9], and influenza outbreaks can occur even in settings with 99% vaccination coverage [10]. Even though the early antiviral treatment of influenza also reduces the probability of influenza-associated complications and mortality [11], antiviral treatment is often infrequently prescribed in outpatient settings because of a lack of timely diagnostic testing which can be due to patients not seeking treatment on time as well as delays owing to testing procedures [12]. Therefore, it is still necessary to deploy timely and accurate influenza diagnosis, and an improvement in diagnostic sensitivity would also improve influenza surveillance [13]. 

Reverse transcription-polymerase chain reaction (RT-PCR) assays, which consistently demonstrate high diagnostic sensitivity, are viewed as one of the “gold standards” of influenza diagnostic methods [14,15,16]. However, PCR is not appropriate for point-of-care (POC) deployment since it usually requires trained staff in laboratories equipped with specialized thermal cycling equipment and strict environmental conditions to prevent contamination [17,18,19]. Although automated PCR systems are under rapid development which could reduce the turnaround time from hours to < 30 min, the issues of contamination, requirement of trained operators, and high cost of machine and test (about $ 30 ~ > $ 100 per test) still hinder their wide use in POC settings [20,21]. In contrast, rapid influenza diagnostic tests (RIDTs), which are antigen-antibody-based lateral flow immunoassays (LFAs), can be completed without skilled technologists in less than 30 min at a lower cost (about < $15 per test and can be even cheaper), and their results can be observed visually in the POC setting [14]. Further, RIDTs are approximately 20–50 times less expensive than PCR tests. As a result, RIDTs are the dominant method for screening influenza infections in POC settings.

However, the current RIDTs implemented in clinics suffer from a low analytical sensitivity, which results in many false negative diagnoses and thus a delay in antiviral treatment and an increase in the spread of the disease. The sensitivity of RIDTs varies between 10% and 70%, although the specificity of the tests is as high as 90% [14]. The CDC also reported that many Food and Drug Administration (FDA)-cleared RIDTs suffer from low sensitivity in the detection of samples with low viral concentrations, thus demonstrating a low overall sensitivity (40%–69%) for all tested specimens [22]. The detection sensitivities of RIDTs also vary by virus type [23]. Even in outbreak settings, the overall sensitivity is not much improved, ranging from 58-79% for different influenza subtypes [24]. 

Numerous efforts have been made to improve the sensitivity of RIDTs by developing novel LFA techniques for POC use. These efforts include assay kinetics optimization and signal amplification in test regions by chemical enhancement and reader use, such as through electrochemical, fluorescence, surface-enhanced Raman scattering, photothermal, and magnetic amplification [25,26]. Several orders of magnitude improvements in detection limits can be achieved with these novel techniques compared with traditional LFAs, as summarized in previous perspective papers [26,27]. 

The thermal contrast amplification (TCA) method was proposed as a photothermal amplification method to improve RIDT sensitivity. Compared with other signal amplification methods, TCA has the significant advantage of simple use. It can be used as a simple and direct add-on step after a commercial LFA without the need to modify or redesign any LFA components or reagents. In TCA, the specifically captured gold nanoparticle (GNP) labels in the test regions are excited by laser irradiation at their plasmon resonance wavelength. This excitation generates strong thermal signals that can be detected by IR sensors and quantified to represent the number of GNPs and, therefore, captured antigens. Our previous studies, as summarized in Table 1, show that TCA can improve the LFAs’ analytical sensitivity by up to 32-fold for commercial LFAs [28,29], and even larger improvements (256-fold) can be achieved when TCA is implemented together with assay optimization and GNP design on LFAs [30].

In real clinical POC use, however, more complicated reaction conditions are expected compared to those in standard antigen-dilution studies. In particular, patient samples can vary widely in viscosity, volume, and range of complex molecules, which may induce the non-specific binding of GNPs in the test region. These factors can impact the LFA performance and thus the TCA outcome. Therefore, a prospective cohort study is needed to evaluate the TCA-LFA diagnostic platform for POC use. Our previous preliminary cohort study [31] reported that the TCA reader was able to identify ~50% of the false negatives from all 88 false negatives in clinical group A: Streptococcus RIDTs (QuickVue Dipstick Strep A Test, Quidel) against the PCR results. In this study, to further evaluate the TCA reader, a double-blind collaborative clinical cohort study was conducted on influenza A and B RIDTs (QuickVue Influenza A + B Test, Quidel) from a larger cohort of patients (*n* = 345) with a local primary care clinic (HealthEast Grand Avenue Clinic, St. Paul, MN, USA). The personnel that operated the TCA reader on the clinical LFAs and confirmatory PCR tests were blind to each other to eliminate potential bias in TCA data analysis and thermal results interpretation. The results show that the TCA reader can substantially improve the sensitivity of the RIDTs (i.e., Quidel LFAs) by visual readout. The improvement in sensitivity achieved by the TCA reader in detecting influenza A was higher than that achieved in detecting influenza B. Although the specificity was compromised slightly by the TCA reader due to the nonspecific binding issues with the LFAs, the overall performance of TCA was still better than that of the visual readout of RIDTs based on comparison of their plots in the receiver operating characteristic space and F1 scores, which is a metric of the accuracy of the diagnostic method. It is also expected that the sensitivity of the TCA reader can be further improved by immediately reading the wet LFAs upon assay completion to eliminate the increase in noise that results from the drying of the LFAs. 

## 2. Materials and Methods

### 2.1. Clinical LFA Samples 

**Figure 1:**
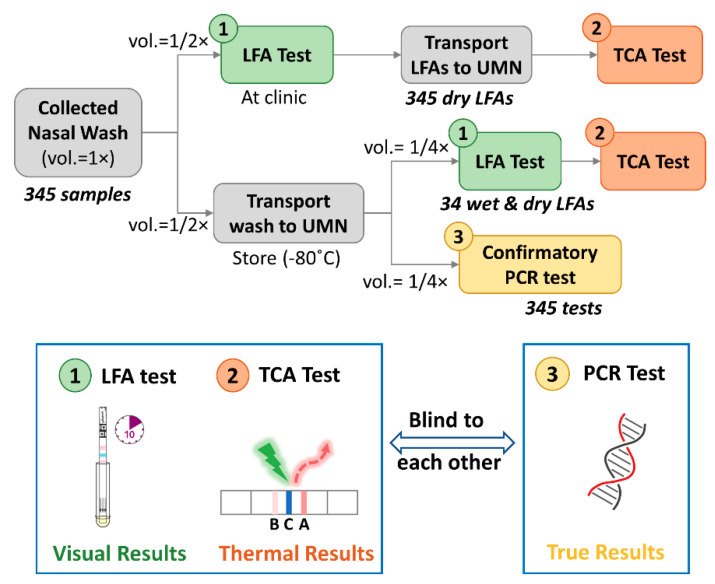
Schematic flowchart of the prospective cohort study. The nasopharyngeal samples were collected (sample volume as 1 unit, i.e., vol. = 1×) and tested by lateral flow assays (LFAs) (available sample vol. = 1/2×) at a local primary care clinic. The remaining wash (further aliquoted into 2 halves, i.e., vol. = 1/2×2) and LFAs were transported to the UMN for confirmative polymerase chain reaction (PCR) tests (available sample vol. = 1/4×) and thermal contrast amplification (TCA) tests (available sample vol. = 1/4×). A comparison of the TCA tests on wet and dry LFAs was conducted on 34 of those wash samples. The operators for the LFA and TCA tests and those for the PCR tests were blind to each other’s results during the experiments. Visual results were recorded after LFA tests while thermal results were obtained from TCA tests. The results from PCR tests were viewed as true results. UMN: University of Minnesota; vol.: volume.

We conducted a prospective cohort study to read commercial LFAs by a TCA reader to study the sensitivity increase of TCA over the standard LFA with visual readout. A schematic flowchart of the cohort study is shown in Figure 1. At HealthEast Primary Care Clinic in St. Paul, Minnesota, we enrolled a prospective cohort of 345 people with suspected influenza illness during the 2017–2018 influenza season. These persons received routine influenza testing via one nasopharyngeal wash per patient. The nasopharyngeal wash was reported to have similar detection sensitivity and to be more comfortable to patients compared to nasopharyngeal swab [35,36,37]. The sample volume from nasopharyngeal wash (~10 mL) can be larger than nasopharyngeal swab (~3 mL) to enable multiple tests by splitting the sample. The collected wash sample was immediately used to perform influenza LFA testing (QuickVue Influenza A + B Test, Quidel, San Diego, CA, USA) at the clinic. After the LFA had been run and the visual readout recorded, the sample, conjugation, and wicking pads were removed from the LFA to stop any further flow and reaction, thus preserving the assay results. The LFA was then placed in a biohazard bag and later transferred to the University of Minnesota for TCA testing. The remaining wash (several mL) was stored at −80 °C and tested in batch for PCR testing via the University of Minnesota Medical Center clinical laboratories. The LFAs and wash samples from the clinic were de-identified from human subjects and coded with a participant identification number. 

### 2.2. Confirmatory and TCA Tests

#### 2.2.1. Confirmatory Reference Standard Test

Each nasopharyngeal wash sample was tested by a confirmatory FDA-approved influenza A/B RT-PCR test (Xpert^®^ Flu/RSV XC assay, Cepheid, Inc., Sunnyvale, CA, USA), the results of which are viewed as the true results (see Figure 1). The correctness (true or false) of the testing readout, such as the visual readout (+/−) of the LFAs at the clinic and the TCA readout (+/−), were determined by comparison with the PCR results (+/−) serving as the reference standard. Each of the tests was performed blinded by different personnel unaware of the other diagnostic test results. 

#### 2.2.2. TCA Test 

As shown in Figure 1, the 345 dry Quidel LFAs collected from the clinic were transported to the UMN and tested with a TCA reader. The protocol for TCA testing followed that of our previous studies [33,38] performed on BD Veritor and QuickVue LFAs for the detection of influenza, malaria, *Clostridium difficile*, and group A: *Streptococcus*. Briefly, the whole region encompassing the test and control lines of the LFA was read by the TCA reader. In analyzing thermal signals, the position of the test line within the tested region was established by the known distance from the control line. The area under the curve (AUC) of the thermal signal within a test line was evaluated as the final thermal signal [33]. An LFA was determined to give a thermal positive or negative readout by TCA by comparing its AUC value to a cutoff threshold, which was set as the summation of the mean and 3 times the standard deviation of thermal signals from 14 PCR-negative samples (true negatives). The diagnostic performance of the TCA readout (+/−) was compared with the PCR results. To avoid any potential bias when performing the TCA tests, the lab personnel running the TCA and PCR tests were blinded to the other’s results. 

Additionally, the effects of the LFA wetness on the TCA readout were also evaluated. Thirty-four remaining wash samples were randomly picked and tested by LFAs (QuickVue Influenza A + B Test, Quidel). Upon completing an LFA and recording its visual readout, the wet LFA was immediately tested by a TCA reader. The LFAs were then stored at least overnight for a later TCA reading on the dry sample. Cutoff thresholds for the wet and dry thermal signals, which determine the TCA positive or negative readout, were obtained by the summation of the mean and 3 times the standard deviation of thermal signals from 4 true negative samples. The diagnostic performance of the TCA readout (+/−) was compared with the PCR results.

## 3. Results and Discussion

**Figure 2:**
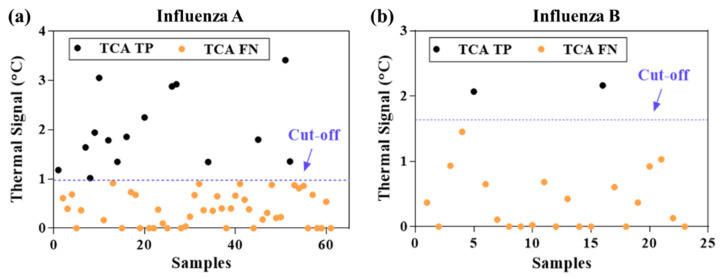
Thermal signals from clinical LFAs that were visually false negatives. (**a**) influenza A: fifteen out of 61 visually false negative samples (~25%) were tested as true positive by TCA. (**b**) influenza B: two out of 23 visually false negative samples (~9%) were tested as true positive by thermal contrast amplification (TCA).

The statistically analyzed TCA results from the 345 Quidel LFAs were compared with those from the visual readout and summarized in Table 2. As can be seen, the sensitivity for both influenza A and B was substantially improved by TCA testing compared with visual readout (influenza A: from 0.32 to 0.49; influenza B: from 0.21 to 0.28). The sensitivity improvement achieved by TCA for influenza A is higher than that achieved for influenza B (relative increase of 53% and 33%, respectively), which is likely due to weaker binding of the GNP labels in the influenza B’s test line or the possibly lower influenza B viral load in the patient cohort compared to that of influenza A. Of note, the sensitivity and specificity of the visual readout of the Quidel LFAs in this cohort study are lower than the claimed values from the manufacturer’s trials (use an FDA-cleared influenza molecular assay as standard results). The claimed sensitivities are 0.815 and 0.809 for influenza A and B, respectively, whereas the specificities are 0.978 and 0.991, respectively [39]. This discrepancy might be caused by a potential difference in manual sample collection and/or percentage of low viral load samples between our cohort study and that carried out by the manufacturer. A similar poor sensitivity with Quidel RIDTs was also reported in a previous cohort study in the 2000-2001 influenza season [16]. Nevertheless, TCA could detect subvisual signals and help compensate for the poor sensitivity of the visual readout to a significant extent (relative improvement of 33–55%). The thermal signals of visual false negative samples are shown in Figure 2. Approximately 25% of the visual false negatives for influenza A can be detected as true positive by TCA and approximately 9% for influenza B. This advantage of TCA to pick out visual false negatives is consistent with our preliminary cohort study for group A: Streptococcus diagnostics [31]. Thus, the results validate the capability of TCA to detect subvisual, weak positives and improve the sensitivity of clinical LFAs. It is also noted that the cutoff value of thermal signals for influenza B in Figure 2b is higher than that for influenza A in Figure 2a. This is likely because the test line of influenza B is at the foremost position in the LFA facing the upcoming flow, thus leading to the maximal possible nonspecific binding of GNPs. 

**Figure 3:**
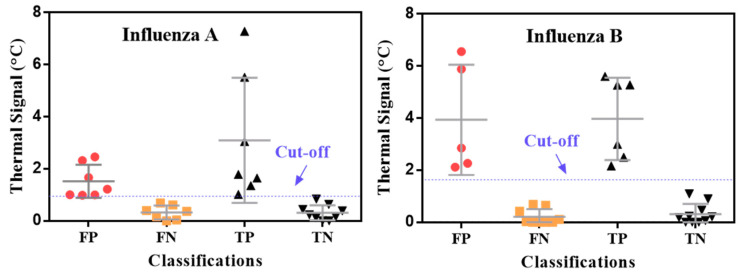
Distribution of thermal contrast signals for influenza A and B. Samples were randomly chosen from 345 clinical LFAs. TP: true positive; TN: true negative; FP: false positive; FN: false negative.

Even though the Quidel RIDTs had a low sensitivity by visual readout, they still had a high specificity (0.99) for both influenza A and B detection (Table 2). Note that TCA can slightly lower the specificity compared with visual readout, as shown in Table 2. However, the final specificities achieved with TCA are still high for influenza A and B detection (0.90 and 0.98, respectively) and comparable to the measured specificities (0.99) and those from the manufacturer’s cohort study (0.97–0.99) [39]. The slight drop in specificity caused by TCA likely stems from the TCA reader amplifying the noise from nonspecifically captured labels at the test lines along with the signal from specifically captured ones. This hypothesis can be proven by inspecting the thermal signals from samples that were randomly selected from the 345 clinical LFAs in Figure 3. This inspection shows clear overlap between the thermal signals from true positives and false negatives. Shifting the cutoff lines could cause either more false positives or more false negatives, which indicates that the limitation is caused by intrinsic nonspecific interactions within the LFA performance. It is also worthwhile to mention that background staining in LFAs can also adversely impact TCA performance. For example, when testing nasopharyngeal wash samples by BD Veritor^TM^ RIDTs (Becton, Dickinson and Company, Sparks, MD, USA), some of these RIDTs showed very strong background staining (see examples in Figure S2) which substantially increased the uncertainty and variation of thermal results from subsequent TCA reading (data not shown here). Therefore, it is inferred that optimizing the assay itself, such as with an improved buffer kit, to reduce nonspecific interactions is critical for improving the specificity of the overall TCA LFA platform. This approach can also increase the signal-to-noise ratio, which in turn enhances the sensitivity [34].

**Figure 4:**
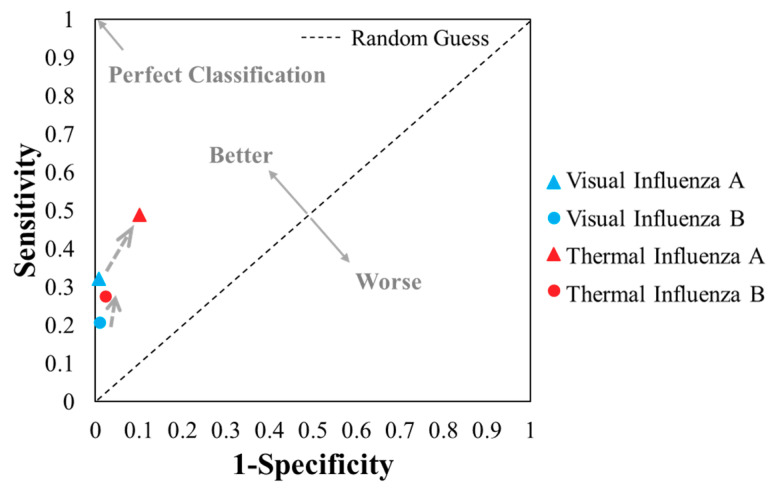
Plots of the 4 results from the visual and thermal contrast amplification (TCA) detection of influenza A and B (from Table 2) in the receiver operating characteristic space.

The overall performance of the TCA readout still surpasses that of the visual readout when comparing their statistical results (from Table 2a–d) in the receiver operating characteristic (ROC) space in Figure 4. Though an informal metric, one point in the ROC space is better than another point if it is closer to the upper left corner, i.e., coordinate (0, 1) [40]. As such, the TCA results showed better performance than the visual results, as their plots are closer to the upper left corner and farther from the diagonal random guess line in Figure 4. Furthermore, the F1 scores, i.e., the harmonic mean of the positive predictive value and true positive rate (Table 2) to indicate a test’s accuracy, of the thermal tests for influenza A and B (0.55 and 0.36, respectively) are also higher than those of visual readout (0.48 and 0.32, respectively for influenza A and B), as shown in Table 2. Although both the accuracy and F1 score are indicators of testing performance, the F1 score is more important in this study due to the imbalanced frequency (i.e., counting) distribution among true positives, true negatives, false positives, and false negatives (Table 2) and the emphasis on false positives and false negatives. In short, the TCA results showed better performance than visual readings in the detection of influenza A and B when tested by Quidel QuickVue LFAs.

**Figure 5:**
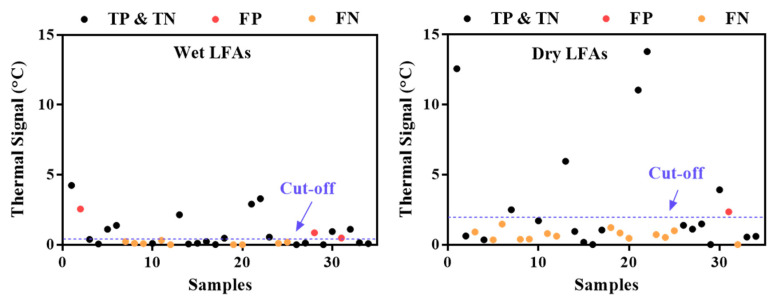
Comparison of thermal signals from wet and dry lateral flow assays (LFAs) from 34 samples. The numbering of the samples is the same in both plots.

TCA readings on wet LFAs from the Quidel RIDTs exhibit better testing performance. The impact of testing a dry vs. a wet LFA on the TCA performance was examined with 34 patients’ nasopharyngeal wash samples, which were randomly chosen for another Quidel LFA test and subsequent TCA scan on both the wet and dry LFAs. The thermal results of the wet and dry samples are compared in Figure 5. Out of the 34 samples, 4 true negative samples were used to determine the cutoff of the thermal signals (i.e., summation of the average thermal signal and 3 times the standard deviation). Due to the extremely imbalanced distribution of true positives, true negatives, false positives, and false negatives for influenza B, only the results for influenza A are presented in Figure 5. The dry LFAs generally have higher thermal signals than the wet ones due to the smaller heat capacity of the membrane when devoid of liquid. However, the uncertainty in the thermal signals also increases, which substantially elevates the cutoff line in the dry results. As a result, more false negatives occurred in the dry results than in the wet results, although some reduction in the number of false positives also occurred. An overall statistical comparison is shown in Table 3. The lower F1 score for the dry results indicates that the dry results are less accurate than the wet results. These results are consistent with our previous findings with group A: Streptococcus LFAs by TCA reading, in which wet LFAs exhibited a slightly higher sensitivity improvement and lower thermal noise than dry LFAs [31]. Thus, we can expect TCA to give better performance when implemented on wet LFAs following assay completion. 

The area of LFA diagnostics has been undergoing rapid change as both assay improvements and sample preamplification strategies along with reader systems are deployed. These approaches are leading to increased sensitivity, speed, and ease of use while maintaining low cost for application in POC diagnostics. [26] Currently, our TCA team is working on reading algorithm improvement, miniaturization, and cost reduction of the TCA reader for eventual commercialization. 

## 4. Conclusions

This prospective cohort study confirms the improved diagnosis of influenza A and B from 345 clinical samples from a prospective clinical cohort by the TCA method, which is a simple add-on step performed after RIDTs. The visual results from RIDTs and thermal results from TCA tests were compared to confirmatory PCR tests, which were viewed as true results. The detection sensitivity of RIDTs was substantially improved by TCA readout (relative increase of 33–55%), although the specificity dropped slightly due to the amplification of nonspecific binding noise from the assay. Overall, the diagnostic performance of the TCA readout surpassed that of the visual readout of RIDTs in terms of the plots in the receiver operating characteristic space and the F1 score. The performance of TCA can be improved by reading wet LFAs upon the completion of the RIDT due to the lower thermal noise in wet LFAs compared to that in dry ones. In summary, TCA is promising for the improved POC diagnosis of infectious diseases with current commercial rapid tests. It is also expected that the TCA performance can be further enhanced by combining TCA with well-designed LFAs having a high signal-to-noise ratio. The translation of laboratory TCA readers to miniaturized commercial readers is ongoing, and further clinical validation with the new readers will be carried out in the future.

## Figures and Tables

**Table 1:** Previous publications to evaluate TCA performance through laboratory dilution studies of pure antigen control samples.

LFAs	Targets	Dilution Sample	Analytical Sensitivity (Improvement, Detection Limit)	Refs
Commercial LFAs	*Cryptococcus*	Single patient human serum	32-fold	[28,29]
	Human hCG	Control solution	20-fold	[32]
	Malaria	Recombinant HRP2 protein and cultured pathogen	4- to 16-fold	[33]
	Influenza A/B	Control swabs	8-fold	
	*C. difficile* GDH	Control solution	8-fold	
	Group A *Streptococcus*	Control solution	4- to 8-fold	[31]
Optimized LFAs	C-Reactive protein (CRP)	Standard human CRP	256-fold, 0.1 ng/mL	[30]
	HIV p24 antigen	Standard p24	8 pg/mL	[34]

hCG: human chorionic gonadotropin; *C. difficile* GDH: *Clostridium difficile* glutamate dehydrogenase.

**Table 2:** Statistical analysis of both the visual and thermal contrast amplification (TCA) readout of clinical lateral flow assays (LFAs) to diagnose influenza A and B. (a) Visual readout of influenza A; (b) TCA readout of influenza A; (c) visual readout of influenza B; (d) TCA readout of influenza B.

		Influenza A				Influenza B			
		(a) Visual		(b) TCA		(c) Visual		(d) TCA	
		TP = 29	FP = 2	TP = 44	FP = 26	TP = 6	FP = 3	TP = 8	FP = 7
		FN = 61	TN = 253	FN = 46	TN = 229	FN = 23	TN = 313	FN = 21	TN = 309
**Sensitivity; TPR**	$\frac{TP}{TP+FN}$	0.32		0.49		0.21		0.28	
**Specificity**	$\frac{TN}{FP+TN}$	0.99		0.90		0.99		0.98	
**FPR**	$\frac{FP}{FP+TN}$	0.01		0.10		0.01		0.02	
**PPV**	$\frac{TP}{TP+FP}$	0.94		0.63		0.67		0.53	
**ACC**	$\frac{TP+TN}{TP+TN+FP+FN}$	0.82		0.79		0.92		0.92	
**F1 score**	$2\times \frac{PPV\times TPR}{PPV+TPR}$	0.48		0.55		0.32		0.36	

TP: true positive; TN: true negative; FP: false positive; FN: false negative; TPR: true positive rate; FPR: false positive rate; PPV: Positive predictive value; ACC: accuracy. Visualization of the relationship between sensitivity and specificity with the classifications from the statistical 2 × 2 matrix can be seen in Figure S1 (Supplementary Material).

**Table 3:** Statistical analysis of thermal contrast amplification (TCA) testing on wet versus dry lateral flow assays (LFAs) for influenza A detection: (a) wet LFAs; (b) dry LFAs.

		(a) Wet LFAs		(b) Dry LFAs	
		TP = 11	FP = 3	TP = 6	FP = 1
		FN = 9	TN = 11	FN = 14	TN = 13
**Sensitivity; TPR**	$\frac{TP}{TP+FN}$	0.55		0.30	
**FPR**	$\frac{FP}{FP+TN}$	0.21		0.07	
**PPV**	$\frac{TP}{TP+FP}$	0.79		0.86	
**ACC**	$\frac{TP+TN}{TP+TN+FP+FN}$	0.65		0.56	
**F1**	$2\times \frac{PPV\times TPR}{PPV+TPR}$	0.65		0.44	

TP: true positive; TN: true negative; FP: false positive; FN: false negative; TPR: true positive rate; FPR: false positive rate; PPV: Positive predictive value; ACC: accuracy. Visualization of the relationship between sensitivity and specificity with the classifications from the statistical 2×2 matrix can be seen in Figure S1.

## Supplementary Materials

The following are available online at https://www.mdpi.com/2075-4418/11/3/462/s1, Figure S1: Relationship between sensitivity and specificity with the classifications from the statistical 2 × 2 matrix. Figure S2: Strong background staining occurred when testing some nasopharyngeal wash samples using BD Veritor^TM^ rapid influenza diagnostic tests (RIDTs).
