# Supplementary material for: Improved Influenza Diagnostics through Thermal Contrast Amplification
Source: Diagnostics (Basel). 2021 Mar 7;11(3):462. doi: 10.3390/diagnostics11030462 (PMC7999055; doi:10.3390/diagnostics11030462)
Supplement: Supplementary file 1 [file diagnostics-11-00462-s001.pdf]

Supporting Information

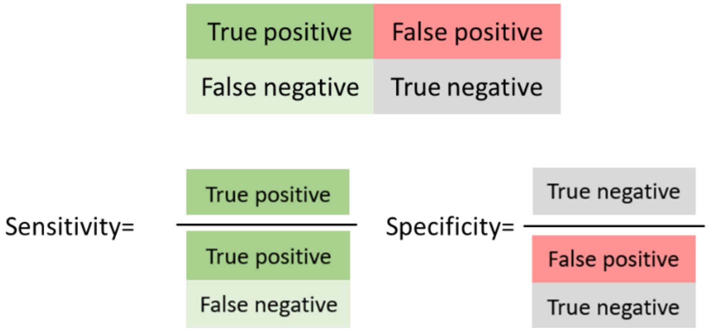

Figure S1. Relationship between sensitivity and specificity with the classifications from the statistical 2×2 matrix.

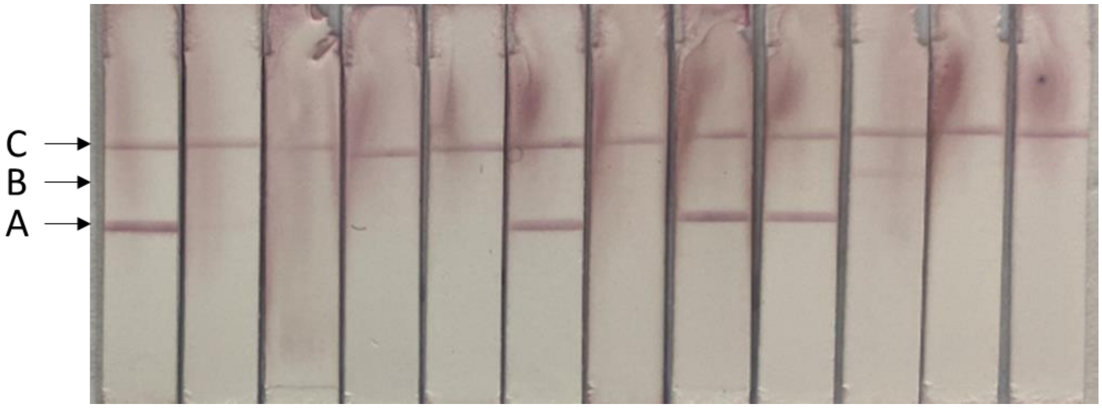

Figure S2. Strong background staining occurred when testing some nasopharyngeal wash samples using BD Veritor^{TM} rapid influenza diagnostic tests (RIDTs). The positions of the control line and test lines for influenza A and B are marked by arrows. C: control line; B: influenza B; A: influenza A.
